# A Novel Multimodal Biometric Person Authentication System Based on ECG and Iris Data

**DOI:** 10.1155/2024/8112209

**Published:** 2024-06-06

**Authors:** K. Ashwini, G. N. Keshava Murthy, S. Raviraja, G. A. Srinidhi

**Affiliations:** ^1^ Siddaganga Institute of Technology, Tumakuru Affiliated to Visvesvaraya Technological University, Belagavi 590018, Karnataka, India; ^2^ University of Garden City, Khartoum, Sudan; ^3^ Sri Siddhartha Institute of Technology, Tumakuru, India

## Abstract

Existing security issues like keys, pins, and passwords employed presently in almost all the fields that have certain limitations like passwords and pins can be easily forgotten; keys can be lost. To overcome such security issues, new biometric features have shown outstanding improvements in authentication systems as a result of significant developments in biological digital signal processing. Currently, the multimodal authentications have gained huge attention in biometric systems which can be either behavioural or physiological. A biometric system with multimodality club data from many biometric modalities increases each biometric system's performance and makes it more resistant to spoof attempts. Apart from electrocardiogram (ECG) and iris, there are a lot of other biometric traits that can be captured from the human body. They include face, fingerprint, gait, keystroke dynamics, voice, DNA, palm vein, and hand geometry recognition. Electrocardiograms (ECG) have recently been employed in unimodal and multimodal biometric recognition systems as a novel biometric technology. When compared to other biometric approaches, ECG has the intrinsic quality of a person's liveness, making it difficult to fake. Similarly, the iris also plays an important role in biometric authentication. Based on these assumptions, we present a multimodal biometric person authentication system. The projected method includes preprocessing, segmentation, feature extraction, feature fusion, and ensemble classifier where majority voting is presented to obtain the final outcome. The comparative analysis shows the overall performance as 96.55%, 96.2%, 96.2%, 96.5%, and 95.65% in terms of precision, *F*1-score, sensitivity, specificity, and accuracy.

## 1. Overview

Since the last 20 years, researchers have noticed an incredible development in the usage of digital data in the forms of audio, video, text, images, and various other types of raw and unprocessed data. This growth is accelerated by the extensive use of development of Internet-based devices. But the growing popularity of these gadgets brought with them a number of problems, including processing, data security, and storage capacity. Since there is currently a vast amount of data available online, it is crucial to maintain secure admittance to the data to shield critical information from numerous threats [1].

Biometric authentication is one of the promising techniques to deal with the different types of attacks on the data. Nevertheless, recent technology growths made it easier for hackers to create fraud technologies that can mimic these physical and traditional security methods, such as pins and passwords, that are easily falsified, and keys are frequently misplaced. However, biometric identification, which verifies a person's identity and behavioural traits, has gained popularity in these applications [2]. Unlike legacy methods like passwords and tokens, biometrics cannot be copied, moved, lost, forgotten, altered, or faked [3]. These days, a wide range of industries, including face, fingerprint, iris, gait, palm vein, hand geometry, DNA, keystroke dynamics, and more, use biometric technologies. But thanks to recent technology developments, fraud systems that can impersonate these anatomical and behavioural characteristics have been created by hackers [4].

Thus, these techniques become vulnerable to various spoofing attacks. Moreover, the present studies have stated that the conventional unimodal or single biometric authentication system may be unreliable because the characteristics of a single biometric system can be contaminated which may lead to the failure of security systems to certain threats [5].

It is simple to counterfeit a single biometric attribute for use. For instance, it is simple to duplicate a fingerprint by employing a finger's fictional ridge pattern. Furthermore, when only one sensor is utilized, a person's face can be faked utilizing neural texture algorithms or deepfake recognition. However, forging many data points will be extremely difficult when multiple sensors are utilized. Additionally, multiple biometrics can offer widely accepted user acceptance, guaranteed correctness, and spoof proof [2, 6]. Researchers have devised a biometric authentication system using multiple traits to mitigate the nonsecurity challenges associated with unimodal security systems. These systems make use of two or more distinct biometrics, allowing their qualities to be combined to provide a strong attribute set that may be utilized for matching [6]. The multimodal biometric system is more dependable for a range of real-time applications since it can identify people effectively in a variety of situations and is noise-resistant. A variety of combinations are included in the schemes with multimodalities that are currently in use, including ear and iris recognition [7], ECG, fusion of palm, iris, and finger veins [5], and many more [1]. In this study, we have used iris and ECG data for authentication since they are useful in determining a person's lifelessness, while attackers can alter other biometrics like veins, palm prints, and fingerprints. Additionally, each individual has distinct physical traits, which is why each user's ECG signal is unique. In terms of authentication accuracy, recent techniques based on ECG authentication have demonstrated notable performance. Fusion techniques have an impact on multimodal biometric systems' accuracy. At several stages of fusion, including feature, rank, score, sensor, and decision level, these fusion procedures can be applied. Regulating the other fusions can produce results with a notable degree of matching precision, but fusion at the level of sensor is heavily reliant on the acquired data quality such as iris and ECG data.

Feature extraction is a major step in biometric feature extraction. A number of techniques and algorithms have been introduced recently for feature extraction. Wang et al. [8] reported that the heart rate is a significant impact on any ECG processing system; thus, the authors focused on the short-term ECG signal identification. Specifically, the short-term ECG signals have fewer readings of heart rates because during verification, the ECG signal is acquired for a short-term duration. Thus, it becomes a challenging task to match the attributes of ECG signals. In [8], the authors developed a principal component analysis network (PCANet) for feature extraction to identify the potential features. Similarly, Huang et al. [9] focused on the ECG signal processing for biometric authentication system. In this work, the authors used an improved local binary pattern- (LBP-) based feature extraction scheme which helps to extract the latent semantic attributes from LBPs. The obtained semantic LBP features are then processed through collective nonnegative matrix factorization learning process. Moreover, the labels are also incorporated to retain the intra- and intersubject similarities and make it robust against the noise and variations.

Similarly, iris feature extraction and matching are also presented to develop the ECG-based biometric authentication system. As discussed before, the fusion of features has an important part to improve the accuracy in iris verification, and based on this, Prabu et al. [10] proposed a modern method to fuse the biometric characteristics of hand geometry and iris of the users. This hybrid feature fusion scheme used LBP and scale-invariant feature transform (SIFT) features. Finally, learning machine- (LM-) based classifier trains the model. Le-Tien et al. [11] adopted a deep learning-based scheme with modified convolutional neural network and Softmax classifier. This model considers segmentation, normalization, and histogram equalization. Further, the modified convolutional neural network is developed which is obtained by presenting the feature extraction and recognition. The extraction of feature is performed by the Resnet50 method, and these features are added to the completely connected layer for training, and finally, the Softmax layer performs the classification. Currently, the multimodal biometric schemes are adopted widely due to their robust performance to obtain the robust performance proposed by the authors. Ammour et al. [12] used the combination of face and iris data to obtain the attributes for biometric classification. For feature extraction, a 2-dimensional log-Gabor filter which is a multiresolution filter is applied. Later facial features are extracted by applying a wavelet transform called singular spectrum analysis (SSA).

In addition, Aleidan et al. suggested an ensemble strategy based on VGG16 pretrained transfer learning (TL) and long short-term memory (LSTM) to identify individuals using ECG characteristics. A 98.7% accuracy rate was attained using the suggested system [13].

Hezil and Boukrouche proposed a new technique of biometric authentication using human ears and a palm recognition system where these give relevant evidence required for security. Local binary patterns (LBP), Weber local descriptors, and binarized image analysis were used to take up the research work. The authors used IIT Delhi, and IIT2 Ear databases were used for authentication using feature-level fusion technique. The results obtained using these multimodes (ear and palm) provided a significant result [14]. In the paper “Cascade Multimodal Biometric System Using Fingerprint and Iris Patterns,” the authors have used multimodal biometric techniques using fingerprint and iris patterns. CASIA-Iris V1 database and FVC 2000 and 2002 fingerprint database were used to carry out the work. Canny edge detection technique is used to detect the edges of the iris image, and Log-Gabor filter is used for the feature extraction of iris, and the minutiae feature extraction algorithm is used to detect and extract finger features [15]. The results showed a good accuracy of 99.86%, whereas the result, when only the iris was used, was only 99.2%, and when only the fingerprint was used, it was 99.36%, respectively. Walia et al. proposed a robust method of biometric authentication by fusing the iris, finger vein, and fingerprint using score-level fusion technique. The databases used were IITD for iris, VERA for finger vein, and FVC 2006 for fingerprint. Gabor wavelet transform was used to extract the features, and ultimately, the fusion method outperformed with good results [16]. The authors in the paper [17] used a score-level fusion technique to detect fake data. They fused different data in both time and frequency domains. The score-level fusion technique encompassed here is based on nonparametric rules, weighted average, and classification modes. The results obtained showed an impressive result using score-level fusion. The proposed method is based on the geographical data, travel demography, and patients' health. The COVID-19 dataset was used in the study. The result obtained was with an accuracy of 97.2% and an *F*1-score of 0.97 [18]. A unique biometric authentication system dubbed BAED, based on ECG detection, was presented by Allam et al. It uses an LSTM network with a customized activation function and a convolutional neural network (CNN) for biometric authentication. To strengthen the model's trustworthiness, a number of important supportive identification parameters, including FMR, FNMR, FAR, and FRR, were calculated and compared [19]. The authors of the publication [20] suggested a method for enhancing person authentication with real-time iris images. The PCA method has been used to extract the features of the iris. Up to 99.73% accuracy was achieved. Rajasekar et al. proposed a new method of multimodal biometric authentication which can be used in smart city projects where authentication of people is a challenging task. The score-level fusion technique has been applied by the authors to fuse fingerprint and iris data. CASIA iris V3 dataset and the FVC2006 fingerprint dataset were used to obtain an accuracy of 99.8% [21]. In the paper [22], Adamović et al. propose a novel method of person authentication using iris data. The approach is based on a machine learning algorithm. Both MMU and IIT Delhi databases have been used. The method suggested here is helpful in eliminating false acceptance rate and enhances classification accuracy. No wavelet transformation technique is employed here. Rather, classification is done based on numeric features.

The multimodal schemes provide a reliable performance and robustness to several noises and fraudulent technologies. As mentioned before, several stages are included in multimodal biometrics which are considered fusion models such as sensor, feature, score, rank, and decision level. In the proposed work, the focus is on feature-level fusion and decision-level fusion to increase the accuracy of detection. The methodology carried out in this work goes like this:

- Preprocessing phase: this step develops a combined image preprocessing and ECG signal preprocessing phase to increase the data quality
- Segmentation and feature extraction: to develop an efficient approach for ECG signal segmentation where peaks and intervals are detected of ECG signals and various features of iris image are extracted
- Feature fusion module: in this stage, we present a feature fusion approach where ECG and iris features are combined and redundant features are discarded
- Finally, the decision-level fusion method along with the score-level fusion model is presented to get the similarity between ECG and iris inputs

The next portion of the paper discusses the following: Section 2 provides a brief literature review about the existing techniques of multimodal biometric authentication system, Section 3 explains the current model, Section 4 presents the outcome of the proposed approach and comparative analysis with already standing techniques, and finally, Section 5 explains the final comments and future opportunities of the research.

## 2. Literature Review

Many numbers of works are carried out in this area of biometric authentication. The current advancement has reported the superior performance of multimodal over conventional unimodal schemes. In this section, we describe the recent methods of multimodal authentication systems.

Multimodal biometrics is a grouping of numerous methods using a number of sensors. Regouid et al. [7] presented a biometric system with multiple traits where a number of inputs are combined like ECG, iris, and ear to collect the biometric samples of the users. The preprocessing phase consists of segmentation and normalization of ECG iris, and ear signals. In addition, the feature extraction phase consists of a combination of 1D, shifted 1D and 1D-multiresolution local binary patterns (LBP). Along with this, the input signals are transformed into a 1D signal. The obtained signals are categorized by using K-nearest neighbour and radial basis function (RBF) classifiers. In [5], the authors also described the challenges of unimodal systems such as low accuracy and unreliability to prevent the attacks and introduced a fusion model which considers the weighting vote strategy. In [23], Mehraj and Mir used an oriented gradient feature based on a histogram to obtain the handcrafted features. Further, this model uses transfer learning (TL) by using AlexNet, ResNet, DenseNet, and Inception models. Zhang et al. [24] used face and voice data for biometric authentication. The improved local binary pattern feature extraction is applied on data which is of type image, and voice activity detection is used for audio signals. Further, an adaptive fusion scheme is applied which considers renowned attributes from the face and voice data and fuses them to obtain the robust recognition for biometric authentication. Su et al. [25] proposed a biometric technique using finger vein and ECG. This model is based on the fusion of feature which is obtained by applying discriminant correlation analysis (DCA). Jardine et al. [26] used face and ear biometrics for authentication. Rather than using the dataset in its input form, it uses a steerable pyramid transform to decompose data into scales and orientations which is used to obtain the texture features of images. These descriptors are statistical features, directional patterns, and local phase quantization. They are applied to generate the most discriminative texture features. The fused features are classified by applying the K-nearest neighbour classification scheme. Mohan and Ganesan [27] presented multimodal biometric classification using electromyography (EMG) finger vein recognition and hand vein recognition data which are used for person recognition. The fusion scheme uses an optimization strategy by combining elephant herding and deer hunting optimization. The fusion is performed based on the weight factors.

Vyas et al. [28] presented a coding-based method known as bit-transition code which is applied on the grouping palm print modality and iris modality. The Gabor filter is used for preprocessing which generates the symmetric and asymmetric parts. These parts are encoded by applying the encoding. Further, score-level fusion is applied on individual palm and iris data to produce the final decision. Chanukya and Thivakaran [29] presented a preprocessing, feature extraction, and classification-based strategy for multimodal biometrics. The preprocessing step includes median filtering. Further, the shape and texture features are extracted from the enhanced image. Finally, an optimal neural network model is applied whose weights are selected with the help of the firefly optimization algorithm. Hammad and Wang [30] used a method based on convolutional neural networks (CNN) to authenticate people using fingerprint and ECG data. Convolutional neural networks help to obtain the robust features from both ECG and fingerprint and generate the pattern. Later, Q-Gaussian multisupport vector machine (QG-MSVM) classification is applied for authentication.

Previous research [31] has demonstrated the efficacy of the discrete wavelet transform (DWT) in characterizing variations in electrocardiogram (ECG) patterns across different individuals. This study leverages the DWT to decompose ECG signals into multiple scales, each representing a specific level of signal coarseness. These scales serve as an initial feature set for subsequent feature selection. By selectively choosing the scales associated with the QRST complex, it becomes possible to retain identity-related information while minimizing interference effects to the greatest extent achievable. However, this approach lac ks in developing the robust feature extraction module and relies on limited attributes. Dar et al. [32] presented an ECG-based authentication approach which comprise of several stages, including ECG preprocessing, feature extraction, feature reduction, and classifier performance evaluation. The ECG segmentation involves detecting the R-peaks, but the system is not reliant on fiducial detection and avoids excessive computational complexity. Feature extraction combines the discrete wavelet transform (DWT) of the cardiac cycle with heart rate variability- (HRV-) based features. To decrease the dimensionality of the feature set, a best-first search method is employed. The classification stage utilizes random forests as the chosen classifier. In [33], the authors introduced the second order difference plot (SODP) nonlinear analysis technique used for analysing time-series data. It enables the identification of features by statistically analysing the distribution of waves. In this study, second order difference plot (SODP) features were extracted using various quantification methods for human identification based on ECG signals. A novel quantification approach, called logarithmic grid analysis, was introduced specifically for ECG-based human identification using second order difference plot (SODP). In [34], the authors introduced a temporal-frequency autoencoding approach for authentication. The approach begins by transforming the raw data into the wavelet domain, enabling a multilevel time-frequency representation. To remove noise components while preserving identity-related information, a feature selection method built on prior knowledge is proposed and employed to the transformed data. Following that, a stacked sparse autoencoder is employed to learn intrinsic discriminative features from the selected data. Finally, the identification task is accomplished using a Softmax classifier.

The paper [35] has demonstrated the use of IoT in their work of biometric authentication with safety and security using a fingerprint scanner to acquire the user's fingerprint details where the attendance will be saved in the cloud automatically. In the paper [19], the authors proposed a new method of biometric authentication using deep learning using a convolutional neural network (CNN) and a long-term memory network. A customized function was used to evaluate the authenticity of the person. ECG beats are identified based on the R-waves, and later, they are fed to the system with a convolutional neural network (CNN) and a long-term memory network to train the data set, and finally, a decision will be taken.

## 3. The Proposed Methodology

The unimodal has several challenges to authenticate the users; thus, multimodal schemes are introduced recently. However, achieving the noteworthy accuracy remains a challenging task. Here, we introduce a new method for multimodal authentication by developing an innovative feature extraction technique for ECG and iris data. Further, a feature fusion and classifications model are presented to learn the pattern and classify them according to their label.

### 3.1. Iris Feature Extraction

**Figure 1:**
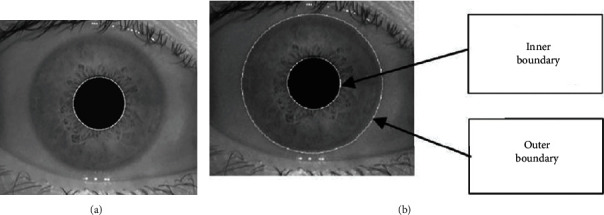
Panel (a) is the internal boundary representation of the iris image, and panel (b) is the external boundary representation of the iris image.

The proposed work has used MMU iris datasets. However, when real-time iris desires to be captured, lighting conditions will not disturb the accuracy as the iris data is captured in a closed chamber which will be having sufficient light requirements. In the first phase, the iris data will be segmented and its features are extracted. Once the iris is segmented, we apply a combination of Gabor filtering and scale-invariant feature transform feature to obtain the robust features from the segmented iris. In order to segment the iris, we first focus on approximating the centre point of the iris; later, inner and outer iris boundaries are identified to segment the desired region as given in Figure 1.

Generally, the iris data capturing devices extract the square region and the said approach here estimates the biggest dark object in the extracted region. To get the segmentation, we initialize from the centre of the image denoted as *P*(*X*_*I*_, *Y*_*I*_). The iterative process is performed in vertical and horizontal directions. This process is iterative and considers a 2 × 2 region from the image centre. Here, we represent the image centre points by  *P*(*X*_0_, *Y*_0_). To find the boundaries of the iris, we focus on finding the radius.

(1) $$\begin{array}{c} r_{s}^{*}=\arg\underset{r\in R}{\min}D_{s}\left(r\right). \end{array}$$

In order to find this, we present the circular edge detector module with the help of convolution operations which inspects the entire image space with the parameter (*x*_0_, *y*_*o*_, *r*) which can be represented as

(2) $$\begin{array}{c} \max_{\left(r,x_{0},y_{0}\right)}\left|G_{\sigma }\left(r\right)*\frac{\partial }{\partial r}\oint \frac{I\left(x,y\right)}{2\pi r}ds\right|. \end{array}$$

**Figure 2:**
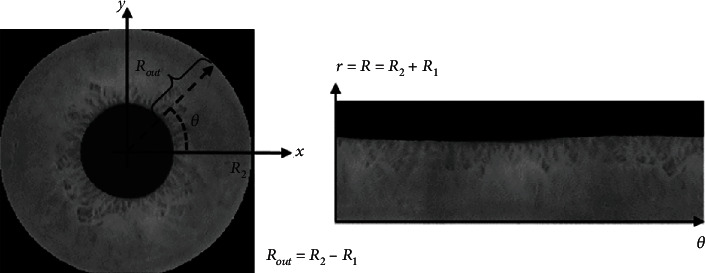
Radius estimation.

This function helps to cover the entire image. Based on this, we obtain the two circular edges which are represented in Figure 2.

**Figure 3:**
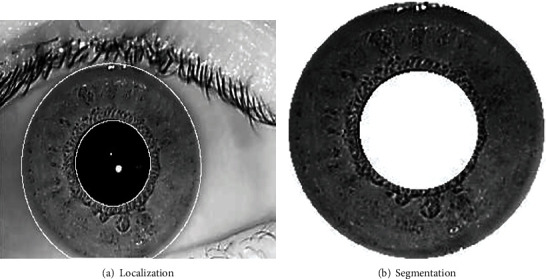
Iris localization and segmentation.

Using this approach, we get the inner and the outer radius of the iris image. Based on this, we crop and segment the iris. The obtained sample outcome is depicted in Figure 3.

**Figure 4:**
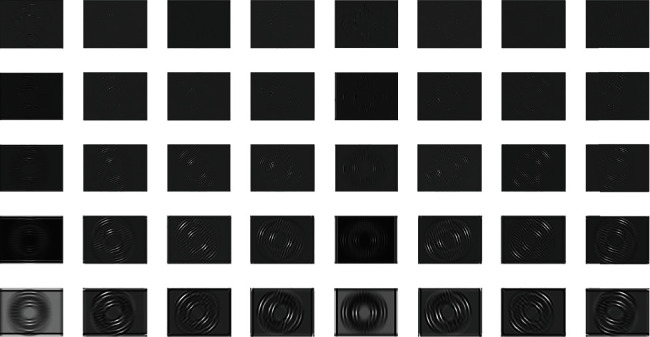
Real parts of Gabor filter-based feature extraction.

The obtained segmented image is used further for feature extraction. We apply combined Gabor and scale-invariant feature transform feature extraction. The transfer function of the one-dimensional Gabor filter revealed in Figure 4 can be expressed as

(3) $$\begin{array}{c} G\left(\omega \right)=\exp\left(\frac{-{\left(\log\left(\omega /\omega_{0}\right)\right)}^{2}}{2{\left(\log\left(\sigma /\omega_{0}\right)\right)}^{2}}\right), \end{array}$$

where *ω*_0_ represents the central frequencies. To improve the robustness of the planned method, we transform the polar coordinates to Cartesian coordinates; thus, the frequency domain form can be expressed as

(4) $$\begin{array}{c} G\left(\omega \right)=\exp\left(\frac{-{\left(\ln\left(u1/f_{0}\right)\right)}^{2}}{2{\left(\log\left(\sigma_{u}/f_{0}\right)\right)}^{2}}\right)*\exp\left(\frac{-v_{1}^{2}}{2\sigma_{v}^{2}}\right), \end{array}$$

where *f*_0_ denotes the central frequency, *σ*_*u*_ is the bandwidth controller for *u*1, *σ*_*v*_ is the bandwidth controller for *v*_1_, and *θ* denotes the orientation of filters.

Further, we extract the odd symmetric function which outperforms when compared with the even symmetric function. The odd symmetric function can be represented as

(5) $$\begin{array}{c} G^{0}\left(\omega \right)=\exp\left(\frac{-{\left(\ln\left(u1/f_{0}\right)\right)}^{2}}{2{\left(\log\left(\sigma_{u}/f_{0}\right)\right)}^{2}}\right)*\exp\left(\frac{-v_{1}^{2}}{2\sigma_{v}^{2}}\right)\sin\left(2\pi f_{0}u1\right). \end{array}$$

**Figure 5:**
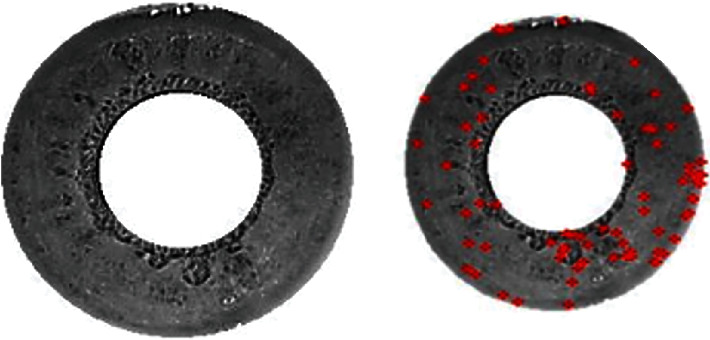
Scale-invariant feature transform detection.

Further, we apply the scale-invariant feature transform (SIFT) feature-based scheme to obtain the scale variation attributes which increases the robustness of attributes irrespective of image acquisition.  Figure 5 depicts the outcome of scale-invariant feature transform detection.

A vast quantity of useful descriptive image features is derived using the SIFT detector. These characteristics are unaffected by scale, rotation, or lighting. These points are usually seen in high-contrast places, potentially on the margins of objects. This helps to generate the robust features from the image irrespective of its rotation, orientation, and scale. The SIFT feature extraction process is as follows:

- In the first step, a scale-space extrema extraction process is applied where interest points of the iris image are extracted for varied scale and scale invariant rotation. This is produced with the help of finding the Gaussian difference function
- In the second stage, a key point localization scheme is performed which is an important part of SIFT. This generates the position and scale for resultant interest points
- The next stage is based on extracting the image gradients and orientation assignment based on these points
- After that, the feature description is carried out. The image gradients with local patterns are measured in the given neighbourhood of a key point at the given scale

### 3.2. ECG Feature Extraction

**Figure 6:**
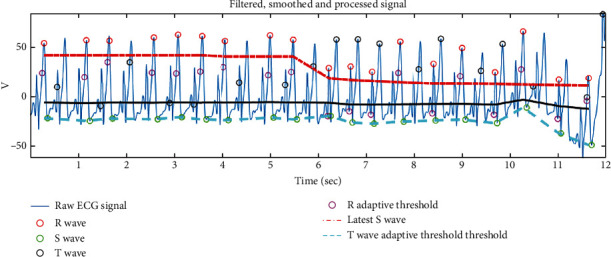
Peak detection in ECG signal.

The proposed multimodal approach considers the iris and ECG data for authentication. The previous section has described the extraction of important features of the iris. In this section, we consider the feature extraction for ECG signals. The proposed approach considers wavelet transform-based feature extraction along with principal component analysis. Moreover, peaks and intervals are detected.  Figure 6 shows the R, S, and T-wave peak detection.

Further, we apply wavelet transform on the input ECG signal to obtain the detailed coefficients. The continuous wavelet transform is expressed as

(6) $$\begin{array}{c} W\left(a,b\right)=\langle x\left(t\right),\psi_{a,b}\left(t\right)\rangle =\frac{1}{\sqrt{a}}\int_{-\infty }^{-\infty }x\left(t\right)\psi^{*}\left(\frac{t-b}{a}\right)\text{dt}. \end{array}$$

Here, *x*(*t*) denotes the original input signal, *ψ*(*t*) is the wavelet basis function, *a* is the dilation, and *b* is the translation factor. With the help of this, we obtain the wavelet transform which is expressed as

(7) $$\begin{array}{c} \psi_{j,k}\left(t\right)=\frac{1}{\sqrt{2^{j}}}\psi \left(2^{-j}t-k\right). \end{array}$$

**Figure 7:**
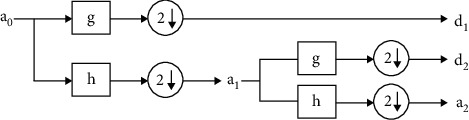
Two-level wavelet decomposition.

In this work, we used the Symlet 8 wavelet function because it generates a more symmetrically supported wavelet which is equivalent to ECG when compared with the other wavelet functions. Further, we perform a 2-level decomposition as depicted in Figure 7.

According to this process, the input original signal *a*_0_ passes through the high-pass filter module *g* and the same signal is processed through the low-pass filter *h*. This stage produces high-frequency subband components and low-frequency subband as *d*_1_ and *a*_1_, respectively. This decomposition process can be expressed as

(8) $$\begin{array}{c} a\left(k,j\right)=\underset{m}{\sum }a\left(m,j-1\right)h\left(m-2k\right),\\ d\left(k,j\right)=\underset{m}{\sum }a\left(m,j-1\right)g\left(m-2k\right). \end{array}$$

Here, *a*(*k*, *j*) and *d*(*k*, *j*) are the *k*^th^ approximation and detailed coefficients, respectively. Later, we applied principal component analysis on the obtained coefficients which is found by finding the covariance matrix *C* from the input data and the mean vector as

(9) $$\begin{array}{c} C=\left(x-\bar{x}\right){\left(x-\bar{x}\right)}^{T}. \end{array}$$

In the next phase, we compute the eigenvalues and eigenvectors from the obtained covariance matrix. Here, the obtained eigenvalues are arranged in largest to smallest order and eigenvectors are arranged as matching to the eigenvalues. Thus, the principal component can be stated as

(10) $$\begin{array}{c} F=l_{j}^{T}x. \end{array}$$

Here, *l* is the arranged vector corresponding to eigenvalues.

### 3.3. Ensemble Classifier

**Figure 8:**
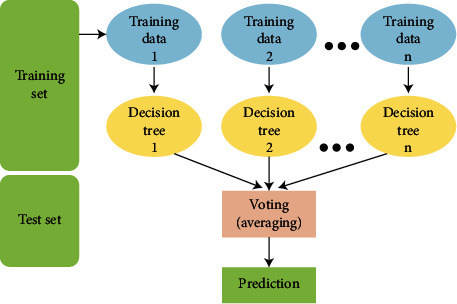
Ensemble classifier.

Here, the final stage of the planned method is proposed where we perform the ensemble classification to learn the patterns from multimodal inputs and predict the final outcome. In order to perform this task, we generate classification trees with the help of a decision tree classifier. Further, the obtained predictions are processed through the majority voting to get the final prediction.  Figure 8 depicts the procedure of the ensemble classifier.

The building of a decision tree depends on the divide-and-conquer process. According to this process, the datasets are trained and contain *T* training data of *k* classes as (*C*_1_, *C*_2_, ⋯, *C* − *k*). During the tree construction process, if *T* consists of a single class then it will be considered as lead. If no cases are present in the *T*, then also, it is a leaf; it will be assigned to a major class of its parent node. On the other hand, if *T* contains a mixed group of classes or a test will be carried out, data *T* will be split into multiple subsets as *T*_1_, *T*_2_, *T*_3_, .., *T*_*n*_. This procedure is repeated until every subset and its belonging class is obtained. Later, we apply the majority voting algorithm to obtain the final predicted output. The majority voting is applied as follows:

(11) $$\begin{array}{c} \hat{y}=\arg\underset{i}{\max}\overset{m}{\underset{j=1}{\sum }}w_{j}X_{A}\left(C_{j}\left(x\right)=i\right), \end{array}$$

where *X*_*A*_ depends on the characteristic function as [*C*_*j*_(*x*) = *i* ∈ *A*] and *A* denotes the unique class label.

## 4. Data Availability Statement and Outcomes

These ECGs are attained from 44 male and 46 female volunteers. The quantity of archives for each user differs from 2 to 20 records. Each signal contains a raw signal and a filtered signal. For this experiment, we have considered 45 user's data from each record and arranged them with their labels. To measure the performance, the dataset is split into 70% for preparation and 30% for verification purpose.

**Figure 9:**
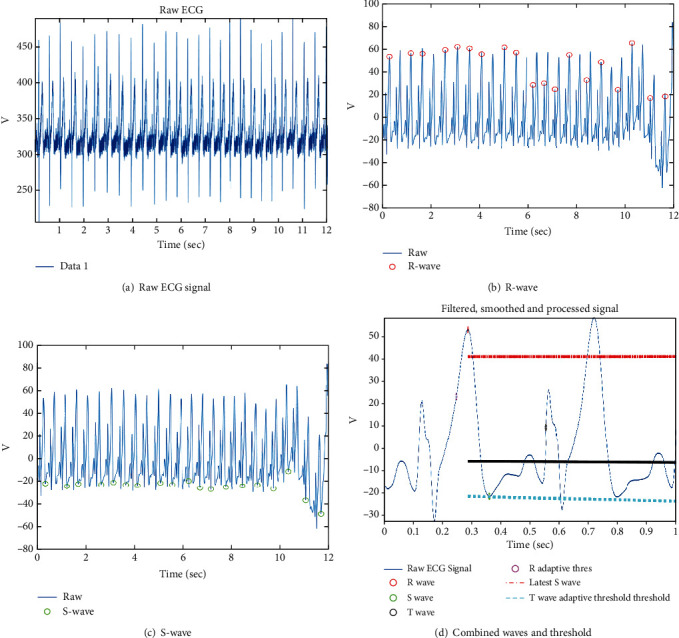
ECG signal processing.

Figure 9 demonstrates a sample outcome of ECG processing where we display the different phases of ECG signal processing. The raw signal is processed through the ECG processing module where signal filtering and various segmentations such as T-wave and S-wave are done.

**Figure 10:**
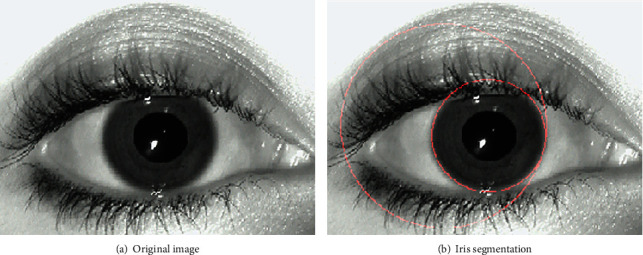
Iris original image and segmented image.

Furthermore, we present iris image processing as represented in Figure 10 where this segmented iris is used for feature extraction.

In this data, 2 class labels are used which are “Genuine” and “Imposter” classes. The ensemble classifiers' performance is measured in accordance with specificity, sensitivity, precision, *F*1-score, and accuracy. The obtained outcome values are linked with the other standard classifiers like bagged ensemble, decision tree, random forest, and proposed ensemble classifiers. For each experiment, we have considered individual and combined biometrics to obtain the classification performance.  Table 1 demonstrates the general representation of the confusion matrix.

**Figure 11:**
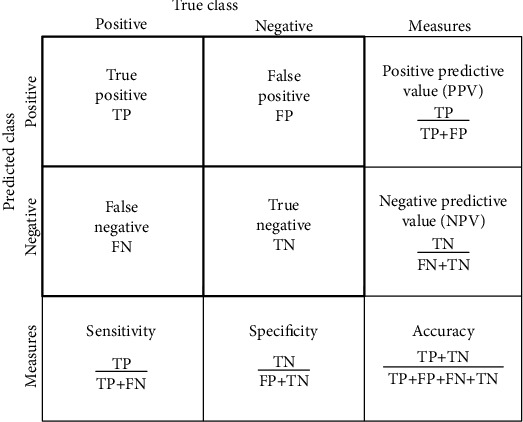
General representation of the confusion matrix.

The confusion matrix of the decision tree classifier is presented in Table 2, and the obtained performance is presented in Figure 11 and Tables 1–4.

Based on these values, we compute the precision, *F*1-score, sensitivity, specificity, and accuracy performance for ECG and iris-based biometric verification scheme as depicted in Table 5. We can observe that the accuracy of the proposed ensemble approach obtained is good compared to random forest at 86.36%, decision tree at 87.50%, and bagged ensemble classifiers at 90.48% which clearly shows the outperformance of the proposed ensemble model.

**Figure 12:**
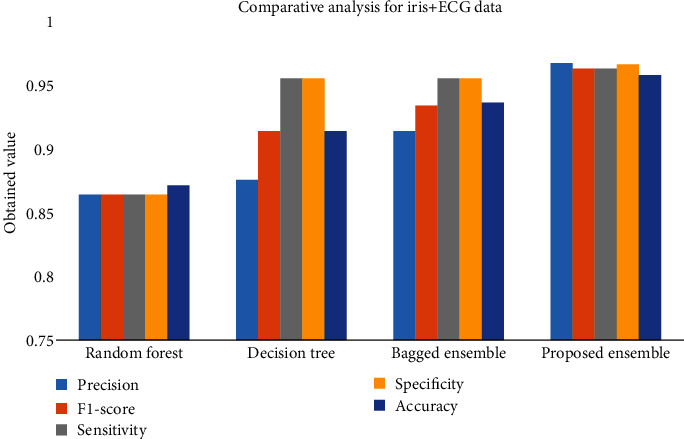
Comparative analysis for iris + ECG data for classifications.

Figure 12 depicts the comparative analysis with respect to accuracy for combined multimodal scenario for different classifiers.

**Figure 13:**
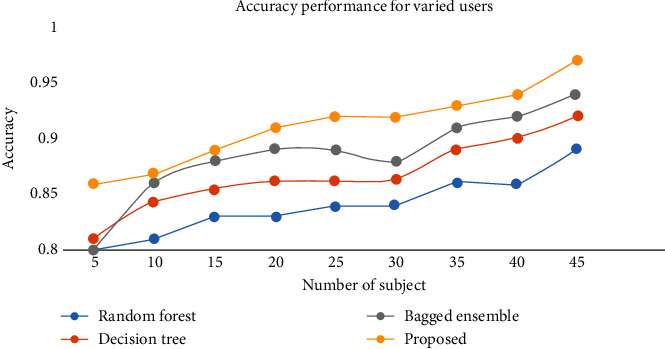
Accuracy comparison for varied users.

The above analysis is compared with other approaches like random forest, decision tree, and bagged ensemble method, and the approach proposed here has a quite interesting performance as the precision score, *F*1-score, sensitivity, specificity, and accuracy, respectively, are 96.55%, 96.2%, 96.2%, 96.5%, and 95.65% for the proposed approach.  Figure 13 shows the accuracy performance with an increased number of subjects.

According to this experiment, we present the comparative analysis in terms of overall verification accuracy, and the average accuracy is obtained as 87%, 84%, 87%, 88.55%, and 94.23% by using decision tree, random forest, bagged ensemble, and proposed ensemble, respectively. The bagged ensemble classifier is mainly used to estimate random sets present in the original database. It further separated the individual predictors required to obtain the final results.

Further, we present a study of comparison for ECG-based authentication systems where the performance of the projected method is compared with existing techniques as stated in [31–33] and [34] as shown in Table 6.

## 5. Conclusion

This paper focuses on the development of an authentication system using multimodalities. The conventional schemes are constructed on the unimodal systems which are not suitable due to poor reliability; thus, currently, multimodal systems are adopted widely in authentication applications. Specifically, ECG plays an important role due to its feature of liveliness detection. Similarly, iris images are important to obtain the unique features. Thus, in this work, we focused on ECG and iris data to come out with an authentication system using multimodalities. The ECG signal and iris data are processed through various phases such as preprocessing and feature extraction (for ECG, we used wavelet and principal component analysis-based morphological features and iris features are extracted by employing Gabor and scale-invariant feature transform (SIFT) feature extraction). Finally, the majority voting-based decision tree ensemble classifier is presented to obtain the final outcome.

## Figures and Tables

**Table 1:** Confusion matrix random forest.

	Genuine	Imposter
Genuine	19 (TP)	3 (FP)
Imposter	3 (FN)	21 (TN)

**Table 2:** Confusion matrix decision tree.

	Genuine	Imposter
Genuine	20 (TP)	2 (FP)
Imposter	2(FN)	22 (TN)

**Table 3:** Confusion matrix bagged ensemble.

	Genuine	Imposter
Genuine	21 (TP)	2 (FP)
Imposter	1 (FN)	22 (TN)

**Table 4:** Confusion matrix proposed ensemble.

	Genuine	Imposter
Genuine	22 (TP)	0 (FP)
Imposter	1 (FN)	23 (TN)

**Table 5:** Performance chart with respect to different classifiers.

Classifier	Precision			*F*1-score			Sensitivity			Specificity			Accuracy		
	Iris	ECG	Combined	Iris	ECG	Combined	Iris	ECG	Combined	Iris	ECG	Combined	Iris	ECG	Combined
Random forest	0.8255	0.8325	0.8636	0.7619	0.8205	0.8636	0.7273	0.7619	0.8636	0.8333	0.9130	0.8750	0.7826	0.8409	0.8696
Decision tree	0.8947	0.9000	0.8750	0.8500	0.9000	0.9130	0.8095	0.9000	0.9545	0.9130	0.9167	0.8750	0.8636	0.9091	0.9130
Bagged ensemble	0.9048	0.9048	0.913	0.9268	0.8837	0.9333	0.9500	0.8636	0.9545	0.9167	0.9167	0.9167	0.9318	0.8913	0.9348
Proposed ensemble	0.9048	0.9091	0.9565	0.8636	0.9091	0.9565	0.8261	0.9091	0.9565	0.9130	0.9167	0.9565	0.8696	0.9130	0.9565

**Table 6:** Comparison of results with respect to the accuracy of an existing system.

Reference	Denoising required	Feature extraction	Classifier	Accuracy
Dar et al. [31]	Yes	Haar	KNN	83.2%
Dar et al. [32]	Yes	Haar	RF	83.9%
Altan et al. [33]	Yes	LGA	SFFS KNN	91.26%
Wang et al. [34]	No	DWT	Softmax	92.3%
Proposed	No	Hybrid of Gabor, SIFT, and wavelets	Ensemble	95.10%

The technique proposed here attains healthier accurateness for ECG-ID database classification because proposed approach uses a combined feature extraction approach to extract the robust features.

## Data Availability

The results of the proposed approach are described here and its comparative analysis with the existing schemes. The said approach is implemented by using MATLAB tool running on Windows 11 platform which has 8 GB RAM and 4 GB NVIDIA graphics card installed. In this experiment, two different datasets MMU iris dataset [36] and ECG ID database from PhysioNet [37] are used in the work. A brief discussion is presented about these datasets: (i) MMU iris dataset (MMU1): This database contains eye images to train the models of iris-based biometric attendance systems. The patterns obtained for each eye are distinctive to each individual, which aids in classifying a person. This dataset contains 460 photos, including 5 shots of each person's left and right iris. This data is available at https://www.kaggle.com/datasets/naureenmohammad/mmu-iris-dataset. (ii) ECG ID database: this database contains a total of 310 recordings of ECG signals captured from 90 people. Each recorded data has the following information: (a) ECG lead I records signal for 20 seconds which is converted to digital form at 500 Hz with 12-bit perseverance. (b) 10 annotated beats. (c) Header (.hea) file which contains the information as age, gender, and date of recording. (d) This data is available in ECG-ID Database v1.0.0 (http://physionet.org).

## References

[1] Rui Z., Yan Z. (2019). A survey on biometric authentication: toward secure and privacy-preserving identification. *IEEE Access*.

[2] Pujari V., Patil R., Sutar S. (2021). Research Paper On Biometrics Security. *Contemporary Research In India*.

[3] Gawande U., Golhar Y., Hajari K. (2017). Biometric-based security system: issues and challenges. *Intelligent Techniques in Signal Processing for Multimedia Security*.

[4] Guelta B., Tlemsani R., Chouraqui S., Benouis M. (2019). An improved behavioral biometric system based on gait and ECG signals. *International Journal of Intelligent Engineering and Systems*.

[5] Zhou C., Huang J., Yang F., Liu Y. (2020). A hybrid fusion model of iris, palm vein and finger vein for multi-biometric recognition system. *Multimedia Tools and Applications*.

[6] Xu H., Qi M., Lu Y. Multimodal biometrics based on convolutional neural network by two-layer fusion.

[7] Regouid M., Touahria M., Benouis M., Costen N. (2019). Multimodal biometric system for ECG, ear and iris recognition based on local descriptors. *Multimedia Tools and Applications*.

[8] Wang D., Si Y., Yang W., Zhang G., Liu T. (2019). A novel heart rate robust method for short-term electrocardiogram biometric identification. *Applied Sciences*.

[9] Huang Y., Yang G., Wang K., Liu H., Yin Y. (2022). Robust multi-feature collective non-negative matrix factorization for ECG biometrics. *Pattern Recognition*.

[10] Prabu S., Lakshmanan M., Mohammed V. N. (2019). A multimodal authentication for biometric recognition system using intelligent hybrid fusion techniques. *Journal of Medical Systems*.

[11] Le-Tien T., Phan-Xuan H., Nguyen-Duy P., Le-Ba L. Iris-based biometric recognition using modified convolutional neural network.

[12] Ammour B., Boubchir L., Bouden T., Ramdani M. (2020). Face–iris multimodal biometric identification system. *Electronics*.

[13] Aleidan A. A., Abbas Q., Daadaa Y. (2023). Biometric-based human identification using ensemble-based technique and ECG signals. *Applied Sciences*.

[14] Hezil N., Boukrouche A. (2017). Multimodal biometric recognition using human ear and palmprint. *IET Biometrics*.

[15] Elhoseny M., Essa E., Elkhateb A., Hassanien A. E., Hamad A. (2018). Cascade multimodal biometric system using fingerprint and iris patterns. *Proceedings of the International Conference on Advanced Intelligent Systems and Informatics 2017*.

[16] Walia G. S., Singh T., Singh K., Verma N. (2019). Robust multimodal biometric system based on optimal score level fusion model. *Expert Systems with Applications*.

[17] Concas S., la Cava S. M., Orrù G. (2022). Analysis of score-level fusion rules for Deepfake detection. *Applied Sciences*.

[18] Gumaei A., Ismail W. N., Rafiul Hassan M. (2022). A decision-level fusion method for COVID-19 patient health prediction. *Big Data Research*.

[19] Jaya Prakash A., Patro K. K., Hammad M., Tadeusiewicz R., Pławiak P. (2022). BAED: a secured biometric authentication system using ECG signal based on deep learning techniques. *Biocybernetics and Biomedical Engineering*.

[20] Hafeez H., Zafar M. N., Abbas C. A., Elahi H., Ali M. O. (2022). Real-time human authentication system based on iris recognition. *Eng*.

[21] Rajasekar V., Predić B., Saracevic M. (2022). Enhanced multimodal biometric recognition approach for smart cities based on an optimized fuzzy genetic algorithm. *Scientific Reports*.

[22] Adamović S., Miškovic V., Maček N. (2020). An efficient novel approach for iris recognition based on stylometric features and machine learning techniques. *Future Generation Computer Systems*.

[23] Mehraj H., Mir A. H. (2021). A multi-biometric system based on multi-level hybrid feature fusion. *Herald of the Russian Academy of Sciences*.

[24] Zhang X., Cheng D., Jia P., Dai Y., Xu X. (2020). An efficient android-based multimodal biometric authentication system with face and voice. *IEEE Access*.

[25] Su K., Yang G., Wu B. (2019). Human identification using finger vein and ECG signals. *Neurocomputing*.

[26] Jardine A., Ramotsoela T. D., Hancke G. P. Biometric authentication system for industrial applications using iris recognition.

[27] Mohan V., Ganesan I. (2021). A nature-inspired meta-heuristic paradigm for person identification using multimodal biometrics. *Concurrency and Computation: Practice and Experience*.

[28] Vyas R., Kanumuri T., Sheoran G., Dubey P. (2022). Accurate feature extraction for multimodal biometrics combining iris and palmprint. *Journal of Ambient Intelligence and Humanized Computing*.

[29] Chanukya P. S., Thivakaran T. K. (2020). Multimodal biometric cryptosystem for human authentication using fingerprint and ear. *Multimedia Tools and Applications*.

[30] Hammad M., Wang K. (2019). Parallel score fusion of ECG and fingerprint for human authentication based on convolution neural network. *Computers & Security*.

[31] Dar M. N., Akram M. U., Usman A., Khan S. A. ECG biometric identification for general population using multiresolution analysis of DWT based features.

[32] Dar M. N., Akram M. U., Shaukat A., Khan M. A. ECG based biometric identification for population with normal and cardiac anomalies using hybrid HRV and DWT features.

[33] Altan G., Kutlu Y., Yeniad M. (2019). ECG based human identification using second order difference plots. *Computer Methods and Programs in Biomedicine*.

[34] Wang D., Si Y., Yang W., Zhang G., Li J. (2019). A novel electrocardiogram biometric identification method based on temporal-frequency autoencoding. *Electronics*.

[35] Jadhav A., Ghodse A., Nahate H., Ghumare S., Kubde R. (2023). Smart attendance monitoring system using biometric. https://ssrn.com/abstract=4382108.

[36] https://www.kaggle.com/datasets/naureenmohammad/mmu-iris-dataset.

[37] https://physionet.org/content/ecgiddb/1.0.0.

